# Pollinator preference and pollen viability mediated by flower color synergistically determine seed set in an Alpine annual herb

**DOI:** 10.1002/ece3.2899

**Published:** 2017-03-22

**Authors:** Junpeng Mu, Yulian Yang, Yanling Luo, Ruijun Su, Karl J. Niklas

**Affiliations:** ^1^ Ecological Security and Protection Key Laboratory of Sichuan ProvinceMianyang Normal UniversityMianyangChina; ^2^ Plant Biology SectionSchool of Integrative Plant ScienceCornell UniversityIthacaNYUSA

**Keywords:** gentianaceae, plant phenology, plant–pollinator interaction, reproductive success, Tibetan Plateau

## Abstract

*Gentiana leucomelaena* manifests dramatic flower color polymorphism, with both blue‐ and white‐flowered individuals (pollinated by flies and bees) both within a population and on an individual plant. Previous studies of this species have shown that pollinator preference and flower temperature change as a function of flower color throughout the flowering season. However, few if any studies have explored the effects of flower color on both pollen viability (mediated by anther temperature) and pollinator preference on reproductive success (seed set) in a population or on individual plants over the course of the entire flowering season. Based on prior observations, we hypothesized that flower color affects both pollen viability (as a function of anther temperature) and pollen deposition (as a function of pollinator preference) to synergistically determine reproductive success during the peak of the flowering season. This hypothesis was tested by field observations and hand pollination experiments in a Tibetan alpine meadow. Generalized linear model and path analyses showed that pollen viability was determined by flower color, flowering season, and anther temperature. Anther temperature correlated positively with pollen viability during the peak of the early flowering season, but negatively affected pollen viability during the peak of the mid‐ to late flowering season. Pollen deposition was determined by flower color, flowering season (early, or mid‐ to late season), and pollen viability. Pollen viability and pollen deposition were affected by flower color that in turn affected seed set across the peak of the flowering season (i.e., when the greatest number of flowers were being pollinated). Hand pollination experiments showed that pollen viability and pollen deposition directly influenced seed set. These data collectively indicate that the preference of pollinators for flower color and pollen viability changed during the flowering season in a manner that optimizes successful reproduction in *G. leucomelaena*. This study is one of a few that have simultaneously considered the effects of both pollen viability and pollen deposition on reproductive success in the same population and on individual plants.

## 1 Introduction

Flower color polymorphism has been long of interest to ecologists and evolutionists (Darwin, 1859; Galen, 1999; Stebbins, 1974). The conveyed wisdom is that pollinators are the primary selective agents driving flower color polymorphisms and flower color transitions (Fenster, Armbruster, Wilson, Dudash, & Thomson, 2004; Malerba & Nattero, 2012; Rausher, 2008; Veiga, Guitián, Guitián, & Guitián, 2015) primarily because pollinators have the ability to perceive and distinguish among different colors. For example, bees have an innate preference for blue flowers, whereas flies have innate color preferences for yellow or white flowers (see Arnold, Savolainen, & Chittka, 2009; Lunau, 2014; Lunau, Wacht, & Chittka, 1996; Raine & Chittka, 2007; Simonds & Plowright, 2004; Thairu & Brunet, 2015). Recent research has also shown that flower color polymorphism within a population or on individual plants can be an adaptation to stressful habitats (Arista, Talavera, Berjano, & Ortiz, 2013; Mu, Li, Niklas, & Sun, 2011; Mu, Li, & Sun, 2010; Rausher, 2008; Schemske & Bierzychudek, 2001). For example, in the case of *Linanthus parryae*, blue‐flowered individuals outperform white‐flowered morphs during years of drought, whereas white individuals survive best during years of high spring precipitation (Schemske & Bierzychudek, 2001). Likewise, in the case of *Parrya nudicaulis*, white‐flowered individuals are survive better than purple‐flowered individuals under cold conditions, whereas purple‐flowered morphs survive best under warm conditions (Dick et al., 2011). There is also evidence that flower color can affect anther temperature and thus pollen viability in the case of some arctic and cold alpine species (Pacini, Frachi, Lisci, & Nepi, 1997; Rao, Jain, & Shivanna, 1992), such as *Gentiana leucomelaena* (Mu et al., 2010). However, to our knowledge, most studies have focused on how either pollinator preference or stressful habitats drive the flower color polymorphisms, either in the same population or on an individual plant, and few if any studies have simultaneously considered the relationships among flower color polymorphism, pollinator preference, floral temperature, and pollen viability at both the level of a population and the level of individual plants.

Theoretically, it is reasonable to suppose that plants in arctic and cold alpine habitats may evolve flower color polymorphisms that simultaneously (1) capitalize on changes in pollinator type and abundance (Fenster et al., 2004; Rausher, 2008) and (2) are adapted to ecological stress induced, for example, by ultraviolet light radiation or fluctuating ambient temperature (Kevan, 1979; Li & Huang, 2009; Mu et al., 2010) over the course of the entire flowering season (Kudo, Nishikawa, & Kosuge, 2004). Indeed, there is nothing that intrinsically precludes the possibility that flower color might change during the flowering season, either at the population level or at the individual plant level, in a manner that adaptively affects floral temperature (e.g., Mølgaard, 1989), pollen viability (Pacini et al., 1997; Rao et al., 1992), and pollinator preference (Li & Huang, 2009; Ollerton, Tarrant, & Winfree, 2011), and in a manner that significantly affects seed set.

**Figure 1:**
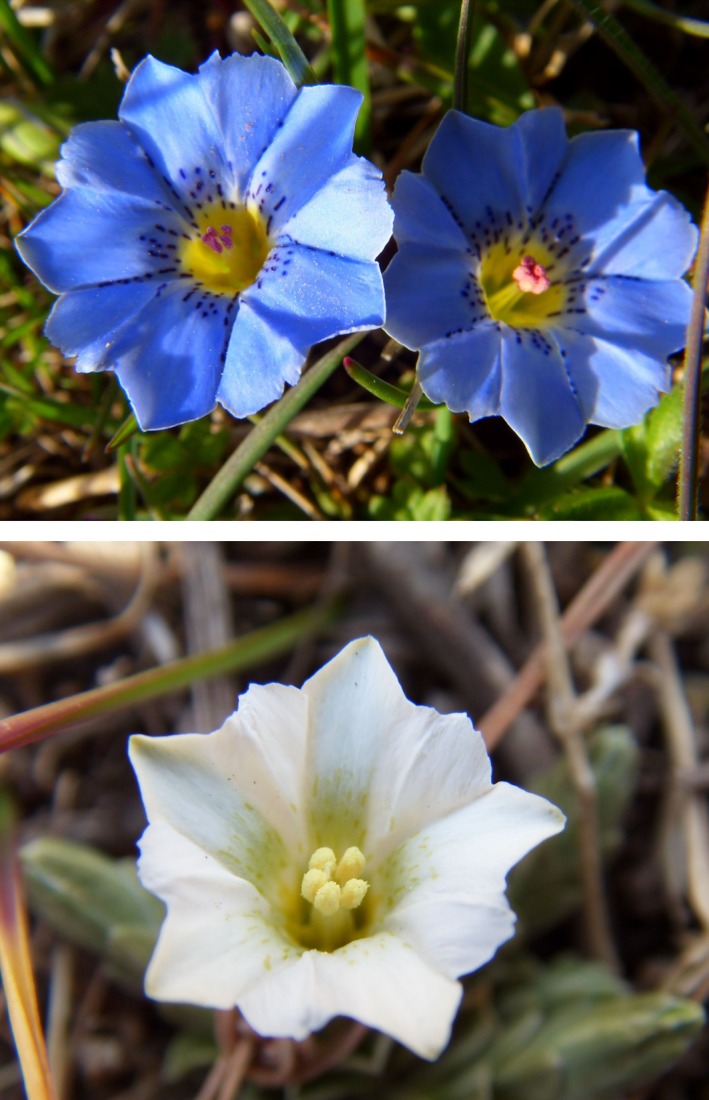
Blue (top) and white (down) flowers of *Gentiana leucomelaena* in an Alpine meadow of Tibet

*Gentiana leucomelaena* provides a model system with which to explore this possibility because flower color polymorphism, pollinator preference and availability, and intrafloral temperature and pollen viability are known to change over the course of the flowering season (Mu et al., 2010, 2011). In addition, this species is an alpine herb that blossoms during the early spring, experiences fluctuating temperatures during the course of the flowering season (Mu, Peng, Niklas, & Sun, 2014; Mu et al., 2010), and produces blue and white flowers within populations and on individual plants (Figure 1). An additional benefit is that each of the two flower color morphs may be either protandrous or protogynous (Mu et al., 2014) and because protandrous flowers tend to outcross, whereas protogynous flowers tend to be self‐pollinated (Bertin, 1993). The species is also useful as a model system because white flowers are more abundant in early Spring when air temperatures fluctuate and when flies (*Calliphora vicina*) tend to be abundant, whereas blue flowers dominate during the peak of the mid‐ to late flowering season (Mu et al., 2011, 2014) when air temperatures are more stable and when bees (*Apis cerana cerana*) are more abundant (Fig. S1; Mu et al., 2011, 2014). Previous studies have also shown that white flowers have higher anther temperatures than blue flowers (approximately 2°C difference; see Mu et al., 2010).

**Figure 2:**
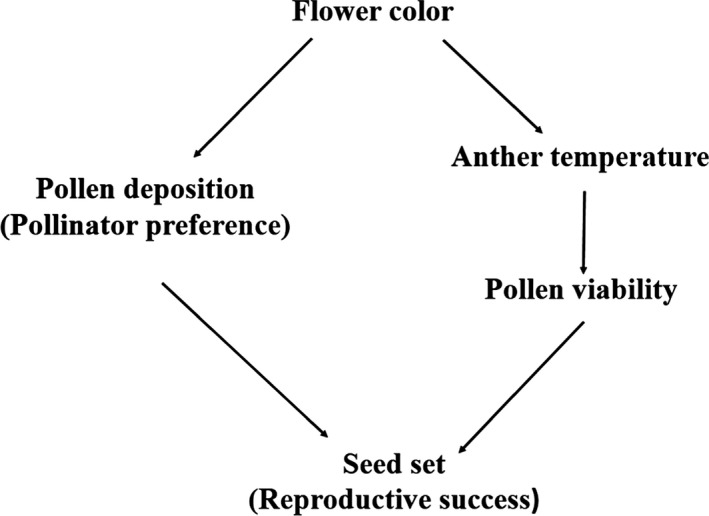
Schematic of the hypothesis that flower color polymorphism affects both pollen viability (through anther temperature) and pollen deposition (through pollinator preference) that jointly affect the reproductive success of *Gentiana leucomelaena*

For all of the aforementioned reasons, the reproductive biology of *G*. *leucomelaena* was used to test the hypothesis that flower color polymorphism affects both pollen viability (through anther temperature) and pollen deposition (through pollinator abundance), which in combination affect reproductive success (Figure 2). Toward this goal, we monitored pollen viability, pollen deposition (number of pollen grains on the pollinator's body and number of pollen grains on stigma), and seed set both under field conditions and hand pollination experiments using protandrous white and blue flowers during the peak of the flowering season.

## 2 Materials and Methods

### 2.1 Study site and model organism

This study was conducted in Hongyuan County, Sichuan Province, which is located in the eastern Tibetan Plateau (32°48′N, 102°33′E). The study site has an altitude of 3,500 m and a cold, continental climate with a long frosty winter and a short and cool spring, summer, and autumn. Detailed information about climate, soil conditions as well as vegetation is presented by Mu et al. (2010, 2011).

*Gentiana leucomelaena* is an alpine annual herb in the Gentiana sect. Chondrophyllae. It grows along streams and in meadows and shrubs in alpine regions of China, with its altitude ranging from 1,900 m (at the eastern edge of the Tibetan Plateau) to 5,000 m. Plants reach 5–10 cm in height and produce from 3 to 15 shoots, each producing one flower, from late March to late May or early June. Fruits mature from late April to mid‐June. The flowering season extends from late March to late May. Specifically, mid‐April to early May are the peak of the flowering seasons (i.e., the time when the maximum number of flowers are being pollinated).

### 2.2 Relationship between anther temperature and pollen viability

To determine the effects of temperature on pollen viability, we monitored anther temperature and pollen development during the peak of the early and mid‐ to late flowering season. Because the flowers opening in March are autogamous, no pollinators visit flowers until mid‐April (Mu et al., 2011). Accordingly, we initiated experiments at mid‐April. We established fifteen 1 × 1 m plots and randomly selected five undamaged plants bearing blue flowers and five undamaged plants bearing white flowers in each plot on April 14. All the plants were tagged using white plastic labels. From each plant, we randomly labeled 2–3 undamaged protandrous flowers that were the first to bloom. There were a total of 200 blue flowers and 200 white flowers, respectively. We then measured anther temperature using an infrared thermometer (AR300, made in Hong Kong Shema, China) three times per 200 flowers from 12:00 to 14:00 each day between April 14 and April 16.

On April 17, we collected pollen grains when anthers began to move toward the petals of each labeled flower (which is indicative of maximum pollen viability based on unpublished data). From each plant, we randomly labeled 1–2 undamaged protandrous flowers. There were a total of 125 blue flowers and 125 white flowers. The pollen was placed into PCR tubes (0.2 ml, made in Shanghai, China), which were labeled to indicate the flower identification number and color, and immediately taken back to the laboratory. Pollen viability was determined using an MTT test solution consisting of a 1% concentration of 2, 5‐diphenyletrazolium bromide (Rodriguez‐Riano & Dafni, 2000). Viable pollen stains deep pink after immersion in this solution, distinguishing it from dead pollen does not stain pink (Rodriguez‐Riano & Dafni, 2000).The viable pollen was counted using a microscope. Between 28 April and May 5, another five plants with blue flowers and five plants with white flowers were labeled within each of the fifteen 1 × 1 m plots. There were a total of 75 plants. Using the same protocols as before, we determined anther temperature and pollen viability of 125 blue protandrous flowers and 125 white protandrous flowers from a total of 150 plants. We calculated the percentage of pollen viability using the protocols of Nepi and Pacini (1993), that is, pollen viability % = [viable pollen number/(viable pollen number + aborted pollen number)] × 100%. All subsequent measurements were made using flowers on the 20 labeled plants per plot (300 in total).

### 2.3 Pollen deposition

The number of pollen grains per pollinator was used as a gauge of pollinator preference. To determine this number, we prepared a safranin dye and a methyl green dye (1% water solution; see Huang & Shi, 2013). As flowers opened under a 2 × 2 × 2 m tent used to exclude pollinators, we used clean forceps dipped into a dye solution to stain pollen within anthers. Within each of the fifteen 1 × 1 m plots, we randomly selected two undamaged protandrous flowers from each of the previously labeled plants. In total, 10 white and 10 blue flowers were selected in each plot; a total of 300 flowers in all of the plots. The pollen grains of white flowers were stained using red safranin dye, and the pollen grains of blue flowers were stained using methyl green dye. Once the anthers had dried, the tent was removed and pollinator behavior was monitored. After a fly or bee visited 5–10 pollen‐dyed flowers, it was collected using electric shock (Huang & Shi, 2013) and placed into a 0.5‐ml PCR tube (made in Shanghai, China). A total of 39 flies were collected during the peak of the early flowering season (between April 15 and 17), and a total of 31 flies and 43 bees were collected during the peak of the mid‐ to late flowering season (between April 30, May 2 and 3). All collections were performed during sunny days because previous experience with the study sites indicated that this was when pollinators were most active. Pollen grain number on each pollinators' body (fly or bee) was examined using a microscope following the protocols of Huang and Shi (2013).

The number of red or blue stained pollen on the stigmas of white and blue flowers was determined in the following way. On April 14, we randomly selected one flower from each labeled plant and emasculated flowers (total of 75 white and 75 blue flowers from all 15 plots) after each had just opened just before anther dehiscence and stigma receptivity (as indicated by the opening of bilobed feather‐like stigmas; see Mu et al., 2011). These flowers were subsequently bagged until stigmas matured on April 18. We then stained the pollen grains of five white and five blue flowers under tents with either safranin dye or methyl green dye for each of the 15 plots. After the stained pollen dried, the tents and bags were removed to permit pollinators access to flowers. After each flower was visited 10 times by one or more pollinators, we placed the stigma into a 0.5‐ml PCR tube. The number of red or blue pollen grains per stigma was subsequently counted using a microscope. A total of 300 flowers were sampled in this manner during the peak of the flowering season (75 white and 75 blue flowers between April 18 and 19, and 75 white and 75 blue flowers between May 2 and 5). All of the flowers were collected on sunny days. Pollen grain number on each stigma was examined using a microscope and the protocols of Huang and Shi (2013). The percentage of red or blue pollen per pollinator or stigma *P*% was calculated using the formula *P*% = [red or blue pollen number/(red pollen number + blue pollen number)] × 100% (see Huang & Shi, 2013).

### 2.4 Seed set under field conditions and under hand pollination experiments

To examine the effects of pollen viability and pollen deposition on the seed set of blue and white flowers under field conditions, we randomly selected two flowers from each labeled plant (i.e., 10 white and 10 blue flowers isolated from each of the 15 plots; a total of 300 flowers) on April 15 and on May 5. Each of the flowers was tagged using white plastic labels recording each plant's serial number and each of the individual flowers that had been labeled per plant. As each fruit matured, it was collected and its seed set was calculated as [seed number/(seed number + aborted seed number)] × 100% (see Mu et al., 2011).

To examine the effects of pollen viability on the seed set of blue and white flowers, we conducted hand pollination experiments from mid‐April to early May. For this purpose, fifteen 1 × 1 m plots were established on 14 April, and 12 flowers (6 white and 6 blue flowers) were randomly selected in each plot. Each flower was labeled and emasculated soon after it opened, but before anther dehiscence and stigma receptivity (Mu et al., 2011). Each flower was then bagged. When stigmas had matured, the white and blue flowers were pollinated with pollen collected from either white or blue flowering plants. Each hand‐pollinated flower was subsequently bagged to prevent a natural pollination event. After fruits ripened, the number of viable and aborted seeds was counted. Seed set was calculated using the protocols of Mu et al. (2011), which were subsequently employed on April 30.

### 2.5 Statistical analysis

We conducted all analyses of fitness using the individual plant mean of traits. Data for each of the traits measured during this study were tested for normality before further analysis. A general linear model was used to assess the effects of anther temperature, pollen viability, pollen deposition, and seed set. For this purpose, four models were established to test the following variables. Model 1 (fixed = anther temperature ~ flower color + flowering season, random = ~1|site) was used to represent a situation where anther temperature is related to flower color and flowering season and their interactions. Model 2 (fixed = pollen viability ~ flower color × flowering season × anther temperature, random = ~1|site) was used to represent the case where pollen viability is related to flower color, flowering season, anther temperature and their interactions. Model 3 (fixed = pollen deposition ~ flower color × flowering season × pollen viability, random = ~1|site) was used to represent the case where pollen deposition is related to flower color, flowering season, pollen viability, and their interactions. Model 4 (fixed = seed set ~ flower color × flowering season × pollen viability × pollen deposition, random = ~1|site) was used to represent the case where seed set is related to flower color, flowering season, pollen viability, pollen deposition and their interactions. Pollen viability was square root‐transformed to normalize model residuals (Waser & Price, 2016). The lowest finite sample‐corrected Akaike information criteria were used to indicate the best model (Sun, Armbruster, & Huang, 2016). Calculations were performed via the glmer function in the lme4 package (Bates, Maechler, & Bolker, 2011), and the glmulti package (Calcagno, 2011) in R (R Development Core Team, 2011).

Based on the hypotheses summarized in the conceptual framework depicted in Figure 2 and the results of best model estimates, a path model was constructed to assess the effects of pollen viability and pollen deposition on seed set and to assess the correlative relationships among flower color, anther temperature, and pollen viability and disposition (Veiga et al., 2015). Seed set was dependent variable and as explanatory variables of flower color, flowering season, anther temperature, anther viability, pollen viability, and their interactions (Veiga et al., 2015). This path model showed that flowering season × flower traits was significant (*p *<* *.05). Accordingly, the data were separated into two phases of the flowering season (the peak of early flowering, mid‐April; the peak of mid‐ to late flowering season, from late April to early May) to perform a more refined and discriminating path analysis (Mitchell, 1992) that included the variables of anther temperature (continuous), pollen viability (continuous), pollen deposition (continuous), seed set (continuous), and flower color (binary). All continuous variables were standardized to a mean of zero and unit variance using the protocols of Maurel, Hanspach, Kühn, Pyšek, and van Kleunen (2016). In this analysis, flower color is a binary trait when using the R package lavaan (http://lavaan.ugent.be/tutorial/cat.html). In addition, path‐parameter estimation methods were used based on maximum likelihood. All path analyses were performed using the R package “lavaan” (Rossel, 2012).

All statistical analyses were performed using R (R Development Core Team, 2015, http://www.R-project.org/).

## 3 Results

### 3.1 Effects of flower color and flowering season on pollen viability

**Figure 3:**
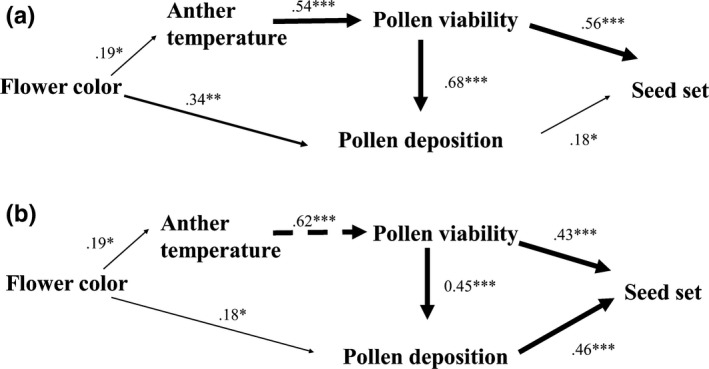
Path diagram showing the relationships among flower color, anther temperature, pollen viability, pollen deposition, and seed set during the peak of the early flowering season (a) and the peak of the mid‐ to late flowering season (b) of *Gentiana leucomelaena*. Line widths indicate the relative strengths of paths. Solid lines represent positive effects, whereas broken lines indicate negative effects. *p <* *.05, **p <* *.01, and ***p <* *.001

**Figure 4:**
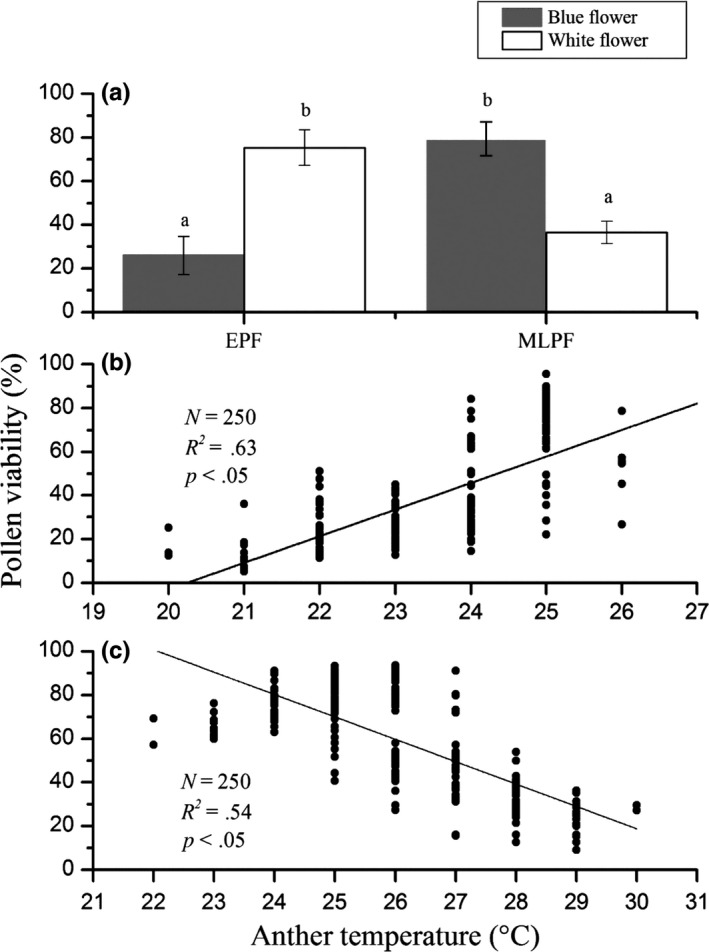
Mean (±*SD*) pollen viability (a) during the peak of the early flowering season (EPF) and the peak of the mid‐ to late flowering season (MLPF) and regression of pollen viability versus anther temperature during the peak of the early flowering season (b) and the peak of the mid‐ to late flowering season (c) that flowers growing under the same conditions. Different letters above columns indicate differences within sites at *p *<* *.05

Flowering season, anther temperature, and their interactions statistically significantly affected pollen viability (Table 1). There was a significant difference in pollen viability changed over the course of the flowering season (Figures 3 and 4). During the peak of the early flowering season (mid‐April), the pollen viability of white flowers was significantly higher than that of blue flowers. In contrast, blue flowers produced more viable pollen during the peak of the mid‐ to late flowering season (from late April to early May, Figure 4). Path analysis showed that the effects of anther temperature on pollen viability changed over the flowering season. Specifically, anther temperature had a positive effect on viability during the peak of the early flowering season (mid‐April) and a negative effect during the peak of the mid‐ to late flowering season (from late April to early May; Figures 3 and 4).

**Table 1:** Summary of the generalized linear models for the effects of flowering season and flower color on pollinator visitation rate, pollen viability, and seed set in *Gentiana leucomelaena*

Determinants of anther temperature		
Best model	Anther temperature = flowering season*** + flower color***	AICc = −1,751.31
Determinants of pollen viability		
Best model	Pollen viability = flowering season*** + flower color + anther temperature*** + flowering season × flower color*** + flowering season × anther temperature***	AICc = 1,780.73
Determinants of pollen deposition		
Best model	Pollen deposition = flowering season*** + flower color** + pollen viability*** + flowering season × flower color***	AICc = 3,717.04
Determinants of seed set		
Best model	Seed set = flowering season*** + flower color + pollen viability*** + pollen deposition***	AICc = 2,047.46

AIC, Akaike information criterion and BIC, Bayesian information criterion. We used model selection to evaluate the models that were lowest finite sample‐corrected, and AIC was used to select the best model (see Sun et al., 2016).

Wald Chi‐square *p*‐values for model‐term effects: ***p <* *.001; **p <* *.01; no asterisk: *p *>* *.05.

Anther temperature was affected by flower color, flowering season, and their interactions (Table 1, Figure 3). The anther temperature of white flowers ranged from 25°C during the early flowering season (mid‐April) to 27°C during the peak of the mid‐ to late flowering season (from late April to early May), whereas the anther temperature of blue flowers ranged from 23 to 25°C during the aforementioned corresponding times. The data showed that pollen viability was significantly reduced when anther temperatures exceeded 26°C or dropped below 24°C regardless of flower color (Figures 3, 4 and S2).

### 3.2 Effects of flower color and flowering season on pollen deposition

**Figure 5:**
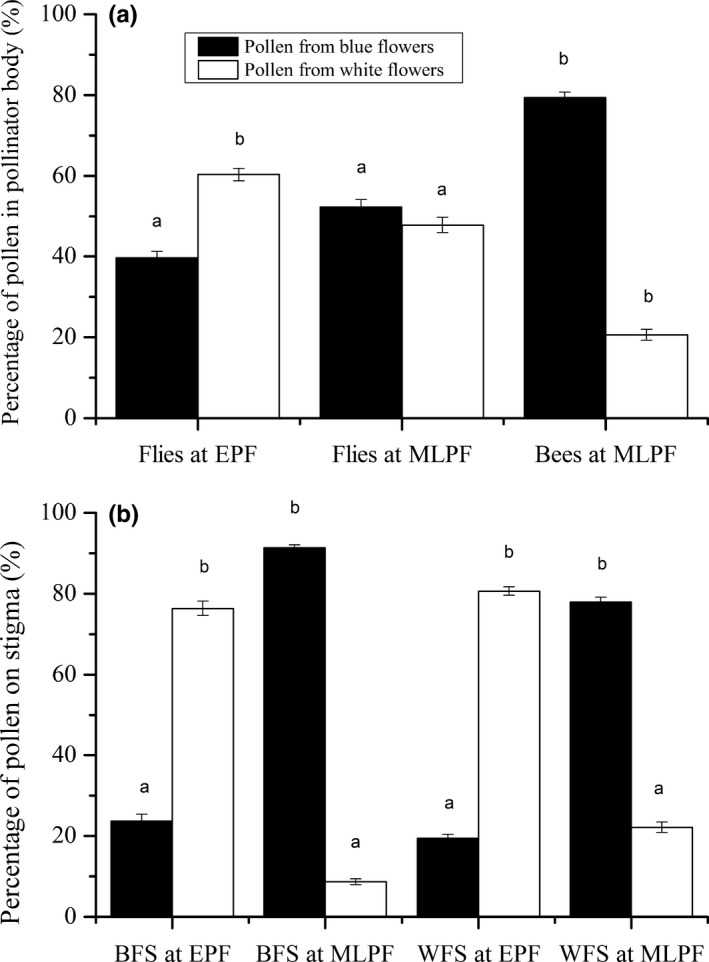
Mean (±SE) percentage of pollen from white and blue flowers on pollinators (a) and stigmas of blue and white flowers (b) during the peak of the early flowering season (EPF) and the peak of the mid‐ to late flowering season (MLPF). Black and white bars denote pollen from blue flowers (pollen grains dyed with methyl green) and pollen from white flowers (pollen grains dyed with safranin). BFS denotes stigmas of blue flowers, and WFS denotes stigmas of white flowers. Different letters above columns indicate differences within sites at *p *<* *.05

Pollen deposition was statistically significantly affected by flower color, flowering season, pollen viability, and the interaction of flower color and flowering season (Table 1, Figure 3). The effectiveness of pollinators on pollen deposition significantly differed between the two flower colors and changed as a function of the flowering season (Figure 5). More red‐strained pollen grains were found on flies and on the stigmas of white and blue flowers during the peak of the early flowering season, whereas more blue‐dyed pollen grains were found on bees and on the stigmas of blue flowers during the peak of the mid‐ to late flowering season. In addition, bees carried more blue‐strained pollen grains than flies throughout the entire flowering season (*t *=* *20.83, *p *<* *.05). Pollen viability was also correlated with pollen deposition throughout the flowering season (Figure 3).

### 3.3 Effects of pollen viability and pollen deposition on seed set

**Figure 6:**
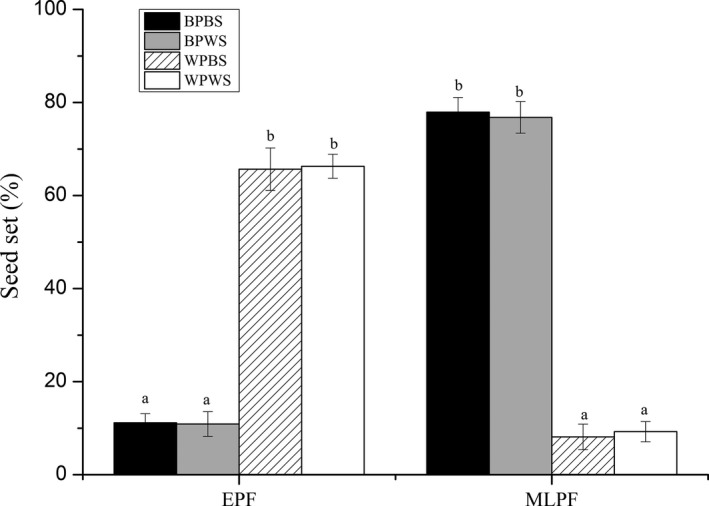
Mean (±SE) seed set after hand pollination using pollen from blue flowers to fertilize the stigmas of blue flowers (BPBS) or white flowers (BPWS), or pollen from white flowers to fertilize the stigmas of blue flowers (WPBS) or white flowers (WPWS) during the peak of the early flowering season (EPF) and the peak of the mid‐ to late flowering season (MLPF) to *Gentiana leucomelaena*. Different letters above columns indicate differences within sites at *p *<* *.05

Seed set was statistically significantly influenced by the phase of the flowering season, pollen viability, and pollen deposition (Table 1, Figure 3). White flowers had significantly greater seed set than blue ones during the peak of the early flowering season, whereas blue flowers had a higher seed set during the peak of the mid‐ to late flowering season. Hand pollination experiments showed that seed set differed between the two flower colors and that it changed over the course of the flowering season (Table 1, Figure 6). Seed set resulting from pollen produced by white flowers was significantly higher during the peak of the early flowering season (mid‐April), but decreased during the peak of the mid‐ to late flowering season (from late April to early May) for both white and blue flowers. Conversely, seed set resulting from pollen produced by blue flowers was significantly lower during the peak of the early flowering season, but dramatically increased during the peak of the mid‐ to late flowering season for both flower colors (Figure 6).

## 4 Discussion

The data reported here show that (1) pollen viability changes with flower color and over the flowering season; (2) pollinator preference also shifts with flower color and over the flowering season (i.e., flies preferentially visit white flowers and carry more white flower pollen during the peak of the early flowering season, whereas bees preferentially visit blue flowers and carry more blue flower pollen during the mid‐ to late flowering season); and (3) pollinator preference and pollen viability conjointly influence reproductive success as measured by viable seed set (Figure 3). These observations support the hypothesis that pollinator preference and pollen viability (as mediated by or at least correlated with flower color) synergistically determine seed set. To the best of our knowledge, these data present detailed example of where the timing of two floral color morphs affects anther temperature and pollinator preference in a manner that influence reproductive success at the level of a population. Specifically, our results show that, during the peak of the early flowering season (when the maximum number of flowers are receptive and when fly activity is at its maximum; mid‐April), white flowers produce more viable pollen grains as a consequence of higher anther temperatures associated with higher visitation rates by flies, which deliver more white flower‐derived pollen grains to blue and white flowers, thereby increasing seed set for both colors of flowers. Subsequently, during the peak of the mid‐ to late flowering season (when the maximum number of flowers are once again receptive and when bee activity is once again at its maximum; from late April to early May), blue flowers maintain an optimal anther temperature and produce more viable pollen grains that are carried by bees to blue flowers, thereby achieving a higher seed set. In summary, the phenological adaptive interactions between different kinds of pollinators and plants with different flower colors throughout the flowering season result in greater reproductive success regardless of whether plants produce white or blue flowers, or both.

The following sections discuss these interactions in more detail.

### 4.1 Effects of flower color and flowering season on pollen viability

Flower temperature is known to have a profound influence on pollen viability (Rao et al., 1992). However, the effects of anther temperature on pollen viability differ among species. Some species require high temperatures to maintain high viability, whereas other species produce viable pollen only when flower temperatures are comparatively low (Issasrakeaisila & Considine, 1994; Rao et al., 1992). In the case of *G. leucomelaena*, both white and blue flowers must achieve and sustain temperatures near 25°C to produce viable pollen (Figures 4 and S2). However, detailed analyses show that a positive relationship exists between anther temperature and pollen viability across temperatures lower than 25°C. In the case of white flowers, optimal anther temperatures are achieved during the peak of the early flowering season with maximum fly activity, whereas blue flowers achieve optimal anther temperatures during the peak of the mid‐ to late flowering season with maximum bee activity. It is noteworthy that the pollen viability of white flowers is dramatically reduced during the peak of mid‐ to late flowering season, owing to an increase in anther temperature, which adversely affects pollen development (Rao et al., 1992). This observation is consistent with those of Djanaguiraman, Prasad, Boyle, and Schapaugh (2013) who report that pollen viability dramatically declines as temperatures approach and exceed 28°C (Figures 4 and S2).

The effect of flower color on floral temperature appears to be an important phenomenon as the anther temperature of white flowers is about 2°C higher than that of blue flowers throughout the flowering season (Mu et al., 2010). This may be attributable to the ability of white petals to reflect 400–800 nm wavelength light (McKee & Richards, 1998) toward immature anthers and therefore increase anther temperatures (Kevan, 1975, 1989; McKee & Richards, 1998). Mølgaard (1989) has observed that *Papaver radicatum* produces white flowers along the north Greenland coast where sunshine is limited, while conspecifics produce yellow flowers more inland and at higher elevations (Mølgaard, 1989), which is consistent with the notion that petal color affects anther temperatures (Kevan, 1989; Mu et al., 2010). Anther color (e.g., yellow vs. pink, see Figure 1) is noticeably different between white and blue flowers and thus may affect anther temperature. Indeed, a previous study shows that anther temperature differs as a function of petal color (e.g., when the blue flowers were painted with red and purple food coloring, the anther temperatures of dyed white flowers tends to be higher than that of dyed blue or purple flowers on sunny days, see Mu et al., 2010), particularly in the case of bowl‐shaped corollas (see Kevan, 1975; McKee & Richards, 1998). Accordingly, there are sufficient data to indicate that flower color and structure can significantly influence intrafloral temperature (Kevan, 1975; McKee & Richards, 1998). Although previous research has shown that humidity is another important factor influencing pollen viability (Khosh‐Khui, Bassiri, & Niknejad, 1976), our measurements reveal no statistically significant difference in humidity between the two color morphs (*t *=* *0.824, *p *=* *.334).

### 4.2 Effects of flower color and flowering season on pollinator preference

Flower color is also a critical trait in the context of pollinator specificity (Asikainen & Mutikainen, 2005; Darwin, 1859). Studies show that bees have good color vision in the ultraviolet, blue, and green, and preferentially visit blue compared to white flowers (Arnold et al., 2009; Ohashi, Makino, & Arikawa, 2015), whereas flies preferentially visit white or yellow flowers (Briscoe & Chittka, 2001). Our data show that flies are the most abundant and active during the peak of the early flowering season, preferentially visit white flowers (Mu et al., 2011, 2014), carry more pollen grains produced by white flowers, and deposit more pollen on the stigmas of white flowers. In contrast, our data show that bees are the most abundant and active during the peak of the mid‐ to late flowering season, preferentially visit blue flowers, carry more pollen grains produced by blue flowers, and deposit them on blue flower stigmas. Although floral morphology is known to influence pollinator preference (Pérez, Arroyo, Medel, & Hershkovitz, 2006; Singer & Sazima, 2001), an examination of the floral morphology of the two color morphs in our study species reveals no statistically significant differences in shape or size (Mu et al., 2011), which leads us to conclude that flower color and the time of flowering during the flowering season are the most critical to determining pollinator preference and pollen deposition.

### 4.3 Effects of pollen viability and pollen deposition on reproductive success

Among entomophilous species, seed production depends on pollen quantity (number of deposited pollen grains per stigma) and pollen quality (pollen viability, see Hove, Mazer, & Ivey, 2016). During the peak of the early flowering season, white flowers produce large quantities of viable pollen that are transported to white flowers by flies. During the peak of the mid‐ to late flowering season, blue flowers produce large quantities of viable pollen that are deposited on the stigmas of blue flowers by bees. This observation is made more significant by the results of hand pollination experiments, which show that seed set dramatically increases when flowers are pollinated with pollen taken from flowers of their corresponding color.

## 5 Conclusions

Our results reveal a dynamic and complex interrelationship among flower color, pollen viability, anther temperature, and pollinator preference that collectively optimizes seed set throughout the flowering season within an especially harsh ecological setting. To the best of our knowledge, this study is one of only a few that has considered the simultaneous effects of all of these variables on seed set within the same population and on the same individual plant. Future study is required to expand our understanding of how other variables, such as air temperature and humidity affect the reproductive success of species such as *G. leucomelaena,* particularly in light of global climate changes and how they affect the reproductive biology of plants currently living in cold habitats.

## Conflict of Interest

None declared.
