# Supplementary material for: Pollinator preference and pollen viability mediated by flower color synergistically determine seed set in an Alpine annual herb
Source: Ecol Evol. 2017 Mar 22;7(9):2947–55. doi: 10.1002/ece3.2899 (PMC5415513; doi:10.1002/ece3.2899)
Supplement: Supplementary file 1 [file ECE3-7-2947-s001.docx]

**Supporting Information**

**Figure S1.** The abundance of fly and bee (A) and visitation rates (B) during the early peak flowering season (EPF) and middle to late peak flowering season (MLPF) of *Gentiana leucomelaena*.

**Figure S2.** Means (±SE) pollen viability for blue and white flowers of *Gentiana leucomelaena* at different temperature treatment. Black and white bars denote blue and white flowers, respectively.

**Figure S1**.

The abundance of flies and bees (A) and mean (± SE) fly and bee visitation rates to blue and white flowers (B) during the early peak flowering season (EPF) and middle to late peak flowering season (MLPF) of *Gentiana leucomelaena*. The abundance of flies and bees was monitored using the methods of Matteson and Langellotto (2009). Pollinator visitation rates were monitored and calculated using the protocols of Arroyo et al. (1985) and Mu et al. (2011). Different letters above columns indicate differences within sites at *P*<0.05.





**Figure S2**

Means (± SE) pollen viability for blue and white flowers of *Gentiana leucomelaena* at different temperature treatments. Black and white bars denote blue and white flowers, respectively. Pollen was collected and pollen viability was tested using the protocols of Hedhly et al. (2005). Different letters above columns indicate differences within sites at *P*<0.05.
